# Minimally Invasive Peroneal Tenodesis Assisted by Peroneal Tendoscopy: Technique and Preliminary Results

**DOI:** 10.3390/medicina60010104

**Published:** 2024-01-05

**Authors:** Rodrigo Simões Castilho, João Murilo Brandão Magalhães, Bruno Peliz Machado Veríssimo, Carlo Perisano, Tommaso Greco, Roberto Zambelli

**Affiliations:** 1 Department of Orthopaedics and Traumatology, Mater Dei Hospital, Belo Horizonte 30170-041, Brazilbrunopmachadov@hotmail.com (B.P.M.V.); zambelliortop@gmail.com (R.Z.); 2 Orthopaedics and Traumatology, Dipartimento di Scienze Dell’invecchiamento, Ortopediche e Reumatologiche Fondazione Policlinico Universitario Agostino Gemelli IRCCS, 00168 Rome, Italy; carlo.perisano@policlinicogemelli.it (C.P.); tommaso.greco01@icatt.it (T.G.); 3 Surgical Department of Faculty of Medical Sciences of Minas Gerais, Belo Horizonte 30170-041, Brazil

**Keywords:** tendoscopy, tenodesis, peroneal tendon, tendon rupture, tendinopathy, endoscopy, foot and ankle, sports injury, minimally invasive surgery

## Abstract

*Introduction:* Peroneal disorders are a common cause of ankle pain and lateral instability and have been described in as much as 77% of patients with lateral ankle instability. Clicking, swelling, pain, and tenderness in the peroneal tendons track are frequent symptoms, but they can be confused with other causes of lateral ankle pain. The management of peroneal disorders can be conservative or surgical. When the conservative treatment fails, surgery is indicated, and open or tendoscopic synovectomy, tubularization, tenodesis or tendon transfers can be performed. The authors present a surgical technique of tendoscopy associated to minimally invasive tenodesis for the treatment of peroneal tendon tears, as well as the preliminary results of patients submitted to this procedure. *Methods:* Four patients with chronic lateral ankle pain who were diagnosed with peroneal brevis pathology were treated between 2020 and 2022 with tendoscopic-assisted minimally invasive synovectomy and tenodesis. Using a 2.7 mm 30° arthroscope and a 3.0 mm shaver blade, the entire length of the peroneus brevis tendon and most parts of the peroneus longus tendon can be assessed within Sammarco’s zones 1 and 2. After the inspection and synovectomy, a minimally invasive tenodesis is performed. *Results:* All patients were evaluated at least six months after surgery. All of them reported improvement in daily activities and in the Foot Function Index (FFI) questionnaire (pre-surgery mean FFI = 23.86%; post-surgery mean FFI = 6.15%), with no soft tissue complications or sural nerve complaints. *Conclusion:* The tendoscopy of the peroneal tendons allows the surgeon to assess their integrity, confirm the extent of the lesion, perform synovectomy, prepare the tendon for tenodesis, and perform it in a safe and minimally invasive way, reducing the risks inherent to the open procedure.

## 1. Introduction

Chronic disorders of the peroneal tendons are a common cause of posterolateral ankle pain, including ankle lateral instability [1]. In a study among professional football players in America, peroneal tendon pathology was found in 4.0% of all ankle injuries [1]. Moreover, peroneal tendon pathology has been described in 23% to 77% of patients with lateral ankle instability [1,2]. It has been estimated that the range for peroneal tendon tears is between 11% and 37% [3,4,5,6,7], and the peroneal brevis tendon is the most involved (88%) [3,8,9]

The peroneal muscles form the lateral compartment of the lower leg. The peroneus longus (PL) muscle becomes tendinous 3 to 4 cm proximal to the distal fibular tip, and the peroneus brevis (PB) muscle usually extends 0.6 to 2 cm more distally [1]. Both muscles receive their innervation from the superficial peroneal nerve and act as the primary evertors of the foot, and both receive their blood supply from the peroneal artery [3].f At the level of the fibular tip, the PB tendon is located anteromedially to the PL tendon, and both share a common fibro-osseous tunnel formed by the superior peroneal retinaculum (SPR), posterolateral fibrocartilaginous ridge and retro malleolar groove within the fibula. This groove was found to be concave-shaped in 82%, flat in 11%, and convex in 7% in a cadaveric study [1]. With contraction, the peroneal longus tendon compresses the brevis against the fibula [3]. Distal to the fibular tip, the tendons become separated by the lateral calcaneal tubercle to enter their own fibrous tunnel, secured by the inferior peroneal retinaculum. This tubercle is considered prominent in 29% of cadaveric specimens, where it can become a source of pain [1].

The mechanism of peroneal tendon injuries has been classically described as a sudden contraction of the peroneal tendons combined with abrupt involuntary dorsiflexion stress of the ankle. However, a plantarflexion and inversion mechanism of injury has also been described in longitudinal ruptures [2].

Clinical presentation is usually by posterolateral ankle swelling, pain, tenderness in the peroneal track, and functional impairment, symptoms that can be confused with other causes of ankle pain [3,4,10]. Passive plantar flexion and inversion of the foot and active plantar flexion and eversion of the foot may provoke tenderness or pain [1,11]. Clicking, subluxation, and luxation of peroneal tendons may also occur [11,12].

It is important to assess the alignment of the hindfoot since the excess valgus can cause a subperoneal impingement of the peroneal tendons, and the excess varus is associated with peroneal tendon pathologies [3,4].

Different diagnoses can emerge from this clinical picture, the most common being tendonitis, tenosynovitis, subluxation or dislocation, and partial and complete peroneal tears [11,13].

Peroneal tendon abnormalities are traditionally investigated using magnetic resonance imaging (MRI). Some anomalies of the peroneal tendons, such as peroneus quartus or low-lying muscle belly on the peroneus brevis, for example, that were not evident or not diagnosed with MRI can be detected using the peroneal tendoscopy [14]. Ultrasound imaging can also be used, with the advantage of dynamic real-time imaging of the peronei, with a 90% accuracy in diagnosing peroneal tendon tears [4].

The management of peroneal pathologies can be conservative, which includes rest, ice therapy, compression, elevation [13], non-steroidal anti-inflammatory drug (NSAID), immobilization [1,4], shockwave therapy [13] and physical therapy [1,11,13]. If this treatment fails, surgery should be indicated, and the procedure is chosen based on the grading of the lesions. Debridement and synovectomy, tubularization, tenodesis, and tendon transfers with auto or allograft reconstruction using an open approach or endoscopic assisted can be indicated [1,5].

If more than 50% of the cross-sectional area of the tendon is involved, some authors suggest tenodesis of the torn tendon to the healthy one [1]; other authors indicate tenodesis if one of the two tendons is torn, and the other is intact [11].

Endoscopic approaches to tendons around the ankle have been described since 1990 [15,16]. An endoscopic procedure would offer several advantages, such as less morbidity, reduction in postoperative pain, and fewer soft tissue complications [16,17,18,19].

The aim of this paper is to present the technique of tendoscopic synovectomy associated to a minimally invasive tenodesis of peroneal tendon tears, and its preliminary results. Our hypothesis is that the minimally invasive peroneal tenodesis provides a good cosmetic result with fewer complications than the traditional open procedure for stenosis. 

## 2. Materials and Methods

After approval of the Institutional Review Board of Ethics in Research, all patients give their informed consent to join the research. Four patients were treated between 2020 and 2022 with this technique, and they were evaluated pre-operatively and at least six months postoperatively. All of them had chronic lateral pain in their ankles, with clinical and imaging (Magnetic Resonance Imaging, MRI) diagnoses of peroneus brevis tendon pathology. All had been submitted to conservative treatment, with medication and physiotherapy, without success. All the procedures were performed by the same two authors using the technique described below. All patients were evaluated at least six months after the operation, and the Foot Function Index (FFI) functional questionnaire was administered by telephone calls [20].

A thorough physical examination was performed on each patient after six months of the surgery. Assessment of edema, scar tissue or scar tenderness, peroneal tendons subluxation or instability, strength, and stabilization capacity were all evaluated at this assessment. Patient standing pattern and gait observation, and all the patients' feelings and opinions regarding the results were registered. This landmark of at least six months was arbitrary, given by the authors to standardize the assessment.

The FFI is a questionnaire developed in English to evaluate foot function in patients who have musculoskeletal injuries. Since evaluation is focused on the foot, the questionnaire has greater accuracy and sensitivity for identifying changes in this area when compared to other available instruments. In assessing the reproducibility of the original FFI, the intraclass correlation coefficient was considered excellent. The questionnaire is divided into blocks of questions: pain and disability, regarding walkability inside the house, need for the use support to walk; difficulty, regarding hardship to walk on different types of floor and ground, climb stairs, walk-in tiptoes, run; pain, in different moments of daily living, such as get up in the morning, walk barefoot, walk with shoes, with orthoses, at the end of the day. All answers are scored, 0 being the least difficulty and 10 being the highest difficulty/limitation [20]. In this research, the highest possible score was 230 points. The more points the patient scores, the greater their disability.

The exclusion criteria were other foot and/or ankle pathologies (instability, arthrosis, fractures), chronic use of corticosteroids, and patient refusal to participate in the research.

### Surgical Technique

**Figure 1:**
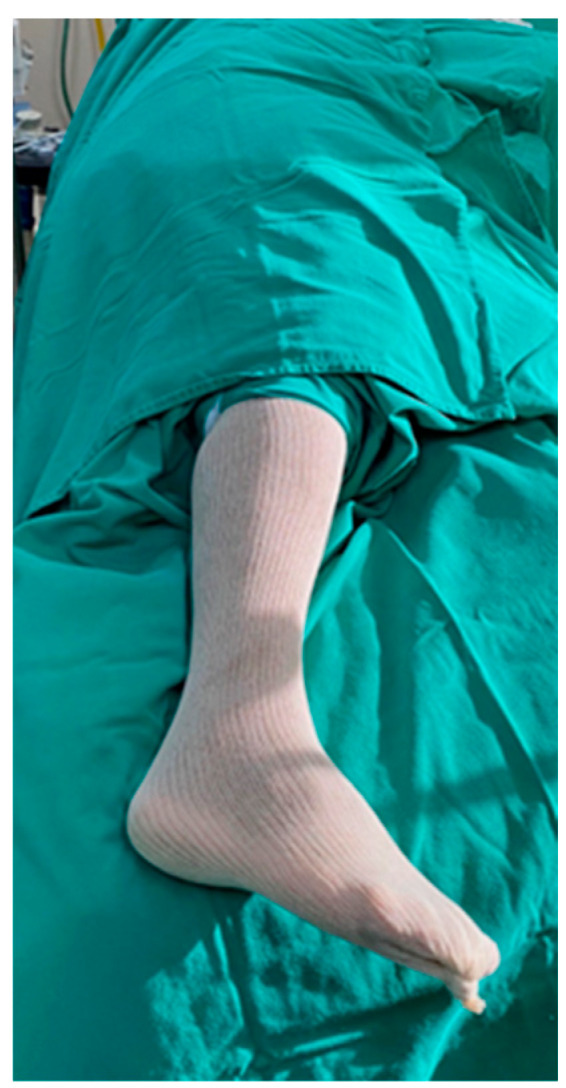
Positioning the patient in lateral decubitus to approach the peroneal tendons.

**Figure 2:**
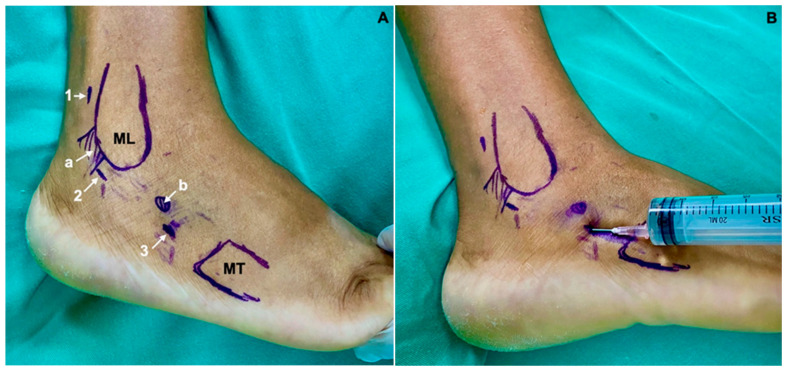
(**A**) Proximal (1), central in the transition of Sammarco’s zones a and b (2) and distal (3) arthroscopic portals. Superior Peroneal Retinaculum (a); Peroneal tubercle (b); lateral malleolus (ML); base of fifth metatarsal (MT). (**B**) Sheath being inflated with 0.9% saline. The distension of the sheath in the path of the peroneal tendons should be noted, avoiding infiltration of the serum outside it.

**Figure 3:**
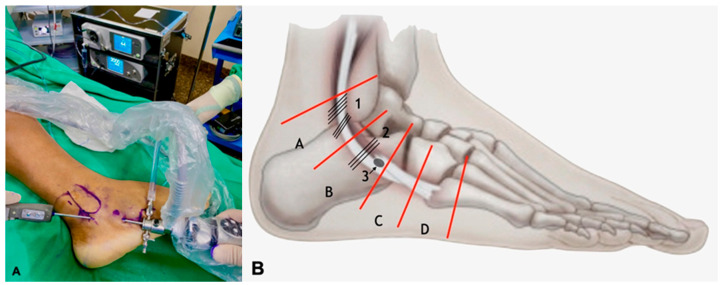
(**A**) Positioning of the arthroscopic instruments for synovectomy in the Sammarco’s zone B. (**B**) Schematic drawing of the Sammarco zones: A, B, C, and D. (1) Superior peroneal retinaculum; (2) Inferior Peroneal Retinaculum; (3) Peroneal tubercle.

The patient is placed in lateral decubitus with the affected limb upwards, with a pneumatic tourniquet at the root of the thigh (Figure 1). The type of anesthesia—spinal anesthesia, peripheric nerve block, sedation, or general anesthesia must be individualized and defined prior to the procedure, considering the clinical characteristics of each patient. In this series, all the patients could be submitted to spinal anesthesia and sedation. The main portals for peroneal tendoscopy are performed directly over the peroneal tendons, 2 cm distal and 2 cm proximal to the distal end of the lateral malleolus. However, they can be performed along the entire length of the tendons. The distal portal is performed first, with skin incision with a scalpel and entry into the sheath using a blunt instrument (trocart). A 2.7 mm arthroscope with a 30° inclination is used for this procedure, and the sheath is inflated with 0.9% saline. The proximal portal is performed under direct vision [16]. In this specific case, three portals were used: one proximal, one in the transition between Sammarco’s zones A and B [10], and another distal, with the aim to allow full access to the entire length of the peroneus brevis tendon (Figure 2A,B). The Sammarco zones are defined as zone A, which comprises the superior retinaculum of the fibularis; zone B, which comprises the inferior peroneal retinaculum to the peroneal tubercle on the lateral calcaneus; zone C, which comprises the bony groove of the cuboid, and zone D, which is distal to the groove up to the insertion of the peroneus longus at the base of the first metatarsal (Figure 3) [10].

**Figure 4:**
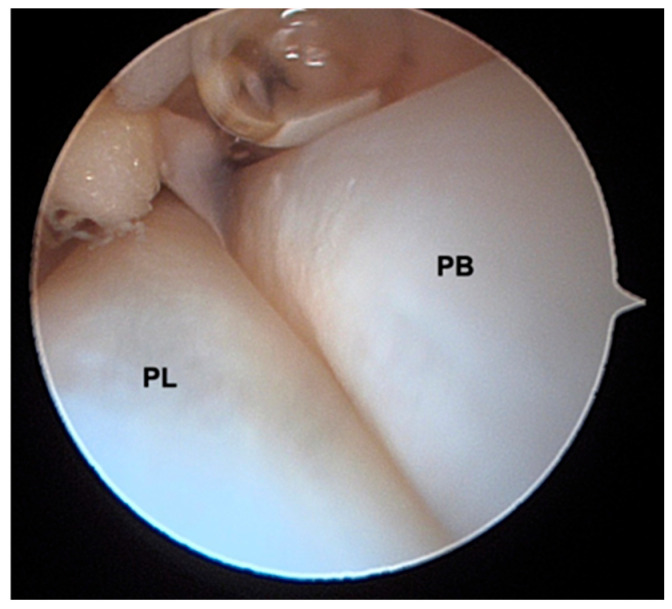
Peroneal Brevis (PB) and Peroneal Longus (PL) position during tendoscopy.

The shaver blade is then introduced, and the synovectomy of both tendons is performed, as well as their inspection (Figure 3 and Figure 4); if a distal implantation of the peroneus brevis musculature is observed, the removal of this musculature must be performed at this time.

**Figure 5:**
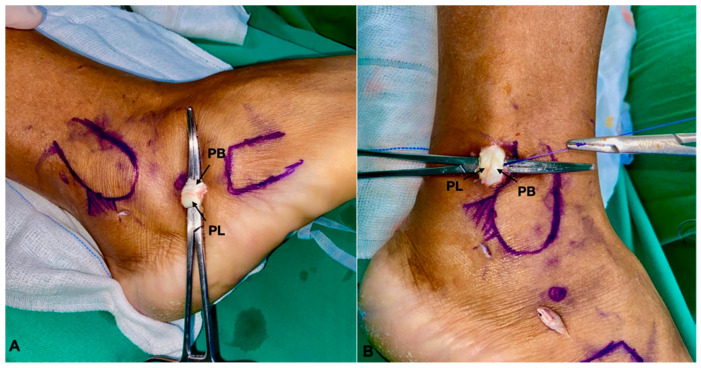
(**A**) Tenodesis is being performed through the distal portal. (**B**) Tenodesis is being performed through the proximal portal. PB: Peroneal Brevis; PL: Peroneal Longus.

After the synovectomy, the arthroscope is removed, and the portals can be enlarged by approximately 1 cm each, through which the peroneal tendons can then be pulled to perform the tenodesis. Using absorbable threads, the tendons are sutured with two “U” stitches distally and two “U” stitches proximally. Next, the tendon to be removed is sectioned under direct view and extracted (Figure 5).

**Figure 6:**
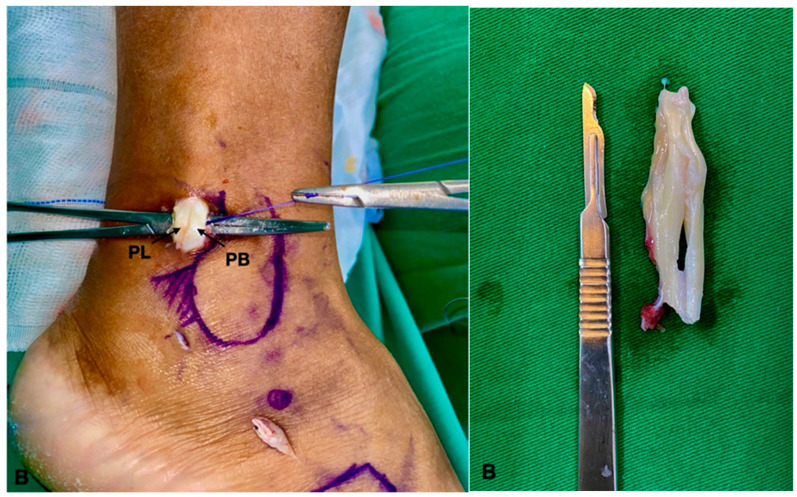
(**A**) Final appearance after the procedure. (**B**) Resected peroneal brevis tendon. PL (peroneal longus); PB (peroneal brevis).

At the end of the procedure, the arthroscope can be reintroduced to review the course and, if necessary, optimize the synovectomy. Subsequently, the accesses are sutured (Figure 6), a sterile compressive dressing is applied, and immobilization is performedg with a plaster splint. In the postoperative period, the splint is kept for two weeks until the stitches are removed. Then, the use of a removable immobilization boot is indicated, with progressive protected weight-bearing and active flexion-extension and intrinsic muscle exercises until the sixth week, when the immobilization is removed, and physical therapy rehabilitation is intensified.

## 3. Results

In the period between 2020 and 2022, four patients underwent peroneal tendoscopy technique associated with minimally invasive tenodesis. The FFI functional questionnaire was applied with pre- and postoperative information to compare the patients’ evolution.

**Figure 7:**
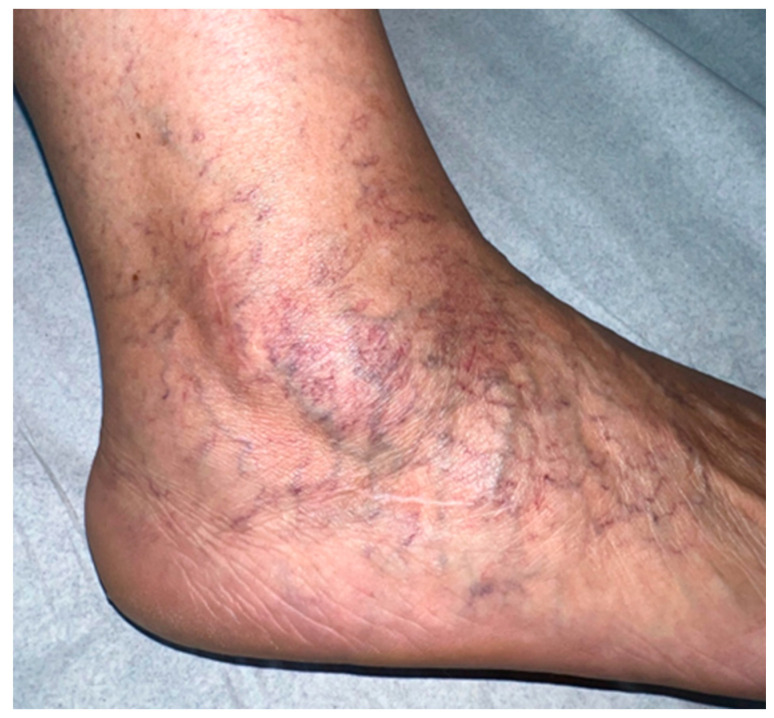
Appearance after 12 months of the procedure.

Patients had no complications related to surgical scars or sural nerves (Figure 7). They reported that they had problems in social activities due to pain and that, after the surgical intervention, they had reduced pain and returned to daily living activities. All of them were satisfied after follow-up with physical therapy rehabilitation and strengthening.

Analyzing the FFI questionnaire, all patients reported an important functional improvement (pre-mean FFI = 23.86%; post-mean FFI = 6.15%), as shown in Table 1. The results were comparable to those obtained in conventional surgery but with less damage to the soft tissues. No skin complications were observed in the operated cases.

## 4. Discussion

Peroneal disorders have historically presented challenges in their effective treatment. Traditionally, they have been performed through a long lateral curved incision from the lateral retromalleolar area right down to the fifth metatarsal’s base. Such a treatment approach, while conventional, wasn’t without its drawbacks. A wide-ranging incidence of complications was reported, with some studies highlighting rates as diverse as 2.4% to 54% [5], such as nerve injuries, infections, postoperative pain, scarring, and stiffness around the ankle joint [18].

Detecting peroneal tendon abnormalities by MRI scan is limited by the quality of the MRI unit and the radiologist’s experience. There are some reports showing that the peroneus quartus muscle and/or peroneus brevis low-lying muscle belly may not be identified in the exam [14].

As medicine evolves, so do its methodologies, driven by innovation, research, and patient-centric approaches. The spotlight is now on minimally invasive procedures, heralding a new era in the surgical treatment of peroneal disorders. At the forefront of this revolutionary transition are the techniques of endoscopic synovectomy and minimally invasive tenodesis. These methods are not just transformative in their approach but have also showcased a marked increase in efficacy. Most patients have reported a noticeable reduction in post-operative discomfort, especially evident six months following the surgical intervention.

Peroneal tendons are good candidates for tendoscopic treatment because of their subcutaneous position along the lateral wall of the calcaneus and posterolateral side of the fibula. This technique allows for a unique view of the entire length of the peroneal tendons while also providing a dynamic evaluation of their movement inside the sheath, and it is a useful tool both for the diagnosis and the minimally invasive treatment of different peroneal tendon disorders [18].

To truly appreciate the significance and intricacies of these novel procedures, we must delve deeper into the specifics, particularly the role of tendoscopy. Visualization, accurate and comprehensive, forms the bedrock of successful surgical outcomes. With advancements in tendoscopy, the entire length and circumference of both peroneal tendons can be visualized from the myotendinous junction proximally to 2 cm proximal to their insertions, using a conventional tendoscopy technique [21]. For visualization of the peroneus brevis tendon, Sammarco zones A and B should be considered. Peroneal brevis tendon tears usually occur in the region of the peroneal sulcus at the distal tip of the fibula at Sammarco zone A [8].

Some authors prefer to perform the proximal portal approximately 3.0–3.5 cm proximal to the tip of the lateral malleolus. It provides surgeons with an expansive operative field during tendoscopy, facilitating ease of entry into the superior posterior retinaculum and providing a larger treatment area during tendoscopy [10,19]. This was also our preference, so a wide synovectomy and resection of the distally implanted muscle belly were performed. Furthermore, it ensures the detailed and careful preparation necessary for tenodesis, all while preserving the superior peroneal retinaculum’s structural integrity.

The advantages of tendoscopy are common to other arthroscopic procedures in ankle surgery. It can be performed as an outpatient procedure or as a “one-day surgery” hospitalization. Surgical morbidity and postoperative pain are reduced when compared to open procedures [18].

Despite the strategic planning, surgical interventions are not devoid of potential challenges. The main disadvantage of this procedure is that it can be technically demanding in patients with extensive tenosynovitis or a scarred/thickened peroneal sheath [18]. Nerve injuries resulting from peroneal tendoscopy are mainly of the sural nerve or its communicating branch with the superficial peroneal nerve in the distal portal [22] or even of the superficial peroneal nerve in the proximal portal, all of which are iatrogenic. In addition, the risk of injury to the sural nerve due to the extravasation of saline solution outside the peroneal sheath has been reported [11,23,24]. In our series, using the endoscopic approach, we haven’t found any nerve complications.

Other complications are reported in the literature. In a study with 30 patients treated for peroneal tendon tears through a long lateral approach, 58% of the patients had scar tenderness, 54% presented lateral ankle swelling, 27% had numbness over the lateral surface of the ankle, and 31% had pain at rest [5].

In a study published in 2020, a case of minimally invasive tenodesis of the peroneus longus tendon was presented [5], but the authors didn’t describe synovectomy during their intervention. In our opinion, synovectomy is a crucial step in the treatment of peroneal tendon pathologies. Several authors cite tenosynovitis as a cause of chronic lateral ankle pain [1,10,11,16,17,18,19,23,24,25,26], thus performing an extensive synovectomy associated with resection of the affected tendon segment and tenodesis from the three steps of the entire treatment of the pathology.

The weaknesses of this study are that it is a case series without a control group to compare the results. Moreover, the number of patients enrolled is still small, and there is a short follow-up period. Prospective comparative studies should be performed to confirm if this combination of procedures is a reliable intervention to treat peroneal tendon disorders.

## 5. Conclusions

The tendoscopy of the peroneal tendons allows the surgeon to assess their integrity, confirm the extent of the lesion, perform synovectomy, prepare the tendon for tenodesis and perform it in a safe and minimally invasive way, lowering the risks related to the open procedure. 

## Figures and Tables

**Table 1:** Pre and postoperative FFI questionnaire results.

Patient	Age	Side	Time (Months)	FFI Pre	FFI Post
1	64	E	23	74/230 (32%)	16/230 (6.9%)
2	51	E	31	27/230 (11.7%)	20/230 (8.6%)
3	60	E	6	60/230 (26.08%)	6/230 (2.6%)
4	60	D	13	59/230 (25.65%)	15/230 (6.52%)
Mean	58.75	3/1	18.25	23.86%	6.15%

## Data Availability

The study data will be available upon request to the corresponding author.
